# Position paper on requirements for toxicological studies in the specific case of radiopharmaceuticals

**DOI:** 10.1186/s41181-016-0004-6

**Published:** 2016-03-21

**Authors:** J. Koziorowski, M. Behe, C. Decristoforo, J. Ballinger, P. Elsinga, V. Ferrari, P. Kolenc Peitl, S. Todde, T. L. Mindt

**Affiliations:** 1 Department of Radiation Physics and Department of Medical and Health Sciences, Linköping University, Linköping, Sweden; 2 Center for Radiopharmaceutical Sciences ETH-PSI-USZ Paul-Scherrer-Institute, 5232 Villigen-PSI, Switzerland; 3 Department of Nuclear Medicine, Medical University Innsbruck, 6020 Innsbruck, Austria; 4 Guy’s and St Thomas’ Hospital, London, SE1 9RT UK; 5 University Medical Center, University of Groningen, 9700 RB Groningen, Netherlands; 6 GE Healthcare, Amersham, UK; 7 University Medical Centre Ljubljana, 1000 Ljubljana, Slovenia; 8 Università di Milano-Bicocca, Tecnomed Foundation, 20090 Monza, Italy; 9 University of Basel Hospital, Radiopharmaceutical Chemistry, 4031 Basel, Switzerland

**Keywords:** Position paper, Pre-clinical toxicology studies, Radiopharmaceuticals

## Abstract

This is a position paper of the Radiopharmacy Committee of the EANM (European Association of Nuclear Medicine) addressing toxicology studies for application of new diagnostic and therapeutic radiopharmaceuticals (RP) that are not approved (i.e., not having a marketing authorization or a monograph in the European Pharmacopoeia), excluding endogenous and ubiquitous substances in humans. This paper discusses the requirements for clinical trials with radiopharmaceuticals for clinical research applications, not necessarily intended to aim at a marketing authorization. If marketing authorization is intended, scientific advice of the competent authorities is mandatory and cannot be replaced by this position paper. The position paper reflects the view of the Radiopharmacy Committee of the EANM and can be used as a basis for discussions with the responsible authorities.

## Background

The existing guideline on toxicology studies applicable to RP “ICH guideline M3(R2) on non-clinical safety studies for the conduct of human clinical trials and marketing authorisation for pharmaceuticals” (CPMP/ICH/286/95) leaves some room for interpretation and is not always clear in describing the type of toxicology study required for various RP applications. Our intention is to give guidance for the specific case of RP and to include other guidelines on impurities in new drug substances “Note for guidance on impurity testing in new drug substances” (CPMP/ICH/2737/99) and genotoxic impurities “Guideline on the limits for genotoxic impurities” (CPMP/SWP/5199/02), “Opinion on use of the threshold of toxicological concern (TTC) approach for human safety assessment of chemical substances with focus on cosmetics and consumer products” (SCCP/1171/08) and FDA’s “Guidance for industry, Developing medical imaging drugs and biological products, Part 1: conducting safety assessments” and FDA’s “Guidance for Industry, Investigators and Reviewers, Exploratory IND studies”. In another important document, “ICH guideline S9 on the nonclinical evaluation of anticancer pharmaceuticals” (CHMP/ICH/646107/08) radiopharmaceuticals are not specifically mentioned, even though oncology is a major field of application for RPs.

## Considerations on preclinical toxicity studies for radiopharmaceuticals

Acute toxicity studies: These are intended to determine the risk of overdosage. Mortality, toxic symptoms and lesions observed during autopsy are registered to determine the no observed effect level (NOEL). This type of study was “traditionally” used as main parameter for toxicity determination before human application of radiopharmaceuticals. Today, it is mainly replaced by so-called extended single dose toxicity studies.

Some type of studies are typically not performed for radiopharmaceuticals these include:

- Subacute and chronic toxicological studies (RP exposure is limited to a single dose or a few doses only)
- Teratogenicity (reproduction) studies (RPs are not given to pregnant women)
- Genotoxic (mutagenic) studies (exposure is limited to a single dose or a few doses only. In certain cases desktop screening (in silico) or the Ames test are sufficient)
- Carcinogenic studies (radiation is a major contributor to cancer induction and this is covered by dosimetric considerations).

For biotechnology-derived products, appropriate nonclinical safety studies should be conducted in accordance with the safety guideline ICH S6 [ICH guideline S6 (R1) – preclinical safety evaluation of biotechnology-derived pharmaceuticals, EMA/CHMP/ICH/731268/1998, European Medicines Agency]. The “Draft guideline on immunogenicity assessment of biotechnology-derived therapeutic proteins” (EMEA/CHMP/BMWP/14327/2006 Rev. 1) may also be applicable.

## Considerations on the compound to be tested for toxicity

Radiopharmaceuticals are prepared by the reaction of a radionuclide with a non-radioactive precursor. Due to the short half-life of most radionuclides employed the preparation is typically done onsite immediately before clinical application. This step is generally performed on the basis of chemical reactions which are adapted in such a way as to meet appropriate radiochemical conditions. There are two basic scenarios:

A) The radiolabelling is characterized by a quantitative reaction of a radionuclide with a chemical precursor, which does not require a purification step. Typically, the precursor is used in large molar excess over the radionuclide (e.g., a complexation agent/ligand over a radiometal). All components, including the precursor (or, e.g., precursor hydrolysis product) and the resulting radiopharmaceutical active ingredient are co-injected.
  In this case, the precursor (or e.g., precursor hydrolysis product) should be used for preclinical toxicity studies. A risk assessment should describe the lowest potential excess of precursor over radionuclide. In the case of doubt, preclinical data comparing the pharmacological activity (e.g., affinity to a receptor) of the precursor and the radiolabelled compound should be provided to support this approach.
B) In cases where radiolabelling reactions are not quantitative, an efficient purification is required to separate the desired radioactive compound from the reaction mixture and ensure low levels of radionuclidic, chemical and/or radiochemical impurities. This involves isolation of the radiopharmaceutical active ingredient, typically by HPLC, and subsequent formulation to the radiopharmaceutical preparation to be injected.
  In these cases the molecule containing a stable instead of a radioactive isotope should be used (e.g., ^19^F instead of ^18^F) for toxicity testing. Data should be provided to show the sufficient separation process of precursor and radiopharmaceutical active ingredient including the mass amount of precursor and radiopharmaceutical active ingredient in the final formulation.

## Proposal: toxicology limits for radiopharmaceuticals

Based on the above outlined considerations and existing guidelines, a new approach for toxicology assessment based upon the definition of three distinct toxicological limits is proposed: 1) <1.5 μg, 2) <100 μg and 3) >100 μg.

### <1.5 μg

This limit is based on the Threshold of Toxicological Concern (TTC) concept. A TTC value of 1.5 μg/day intake of a genotoxic impurity is considered to be associated with an acceptable risk (excess cancer risk of <1 in 100,000 over a lifetime) for most pharmaceuticals. It should be noted that in “Note for guidance on impurities in new drug products” (CPMP/ICH/2738/99) a total daily intake (TDI) of an impurity may be up to 2 mg.

Based on case-by-case judgment for RPs applied in amounts of <1,5 μg injected dose, no toxicology tests may be required, however potential toxicity should be addressed and discussed. The evaluation of potential toxicity may be performed by desktop screening (in silico) and (quantitative) structure-activity relationship (Q)SAR). FDA is considering a tiered approach which ranges from <1,5 μg/person/day as a general threshold, <15 μg/person/day for chemicals without structural alerts for carcinogenicity or with negative mutagenicity test results (Ames test) and <45 μg/person/day for chemicals without structural alerts for carcinogenicity or with negative mutagenicity test results (Ames test) and a LD50 (median lethal dose) >1000 mg/kg bodyweight.

Compounds excluded from this concept: high potency genotoxic carcinogens such as aflatoxin-like-, N -nitroso-, and azoxy-compounds. Risk assessment of members of such groups requires compound-specific toxicity data [Guideline on the limits for genotoxic impurities, CPMP/SWP/5199/02, European Medicines Agency; Opinion on Use of the Threshold of Toxicological Concern (TTC) Approach for Human Safety Assessment of Chemical Substances with focus on Cosmetics and Consumer Products, SCCP/1171/08, European Commission; ICH guideline M7 on assessment and control of DNA reactive (mutagenic) impurities in pharmaceuticals to limit potential carcinogenic risk, EMA/CHMP/ICH/83812/2013, European Medicines Agency]. Any substance with positive Ames test should be evaluated on a case-by-case basis. It must be mentioned that the TTC approach allows up to 120 μg for a single dose in a recent ICH document (ICH M7) soon replacing current guidelines that suggests even room for extension of this concept [Multidisciplinary ICH guidelines including M 7].

### <100 μg

In this case (so-called microdosing) the “Note for guidance on non-clinical safety studies for the conduct of human clinical trials and marketing authorization for pharmaceuticals” (CPMP/ICH/286/95) can be applied.

For <100 μg, approach 1 in “ICH guideline M3(R2) on non-clinical safety studies for the conduct of human clinical trials and marketing authorisation for pharmaceuticals” is applicable. Typically, a 100 x times the clinical dose in thirty animals (rodents) and examination in ten animals/sex on the day following the injection and five animals/sex after 14 days (hematology, clinical chemistry, necropsy, and histopathology) should be used. The in vivo toxicology tests must be performed in compliance with Good Laboratory Practice (GLP), interspecies scaling (allometric scaling) should be applied to calculate from animal to human dose [Note for guidance on non-clinical safety studies for the conduct of human clinical trials and marketing authorization for pharmaceuticals, CPMP/ICH/286/95, European Medicines Agency].

The 1000 x time scaling is mentioned in the same guideline approach and may be followed if the allometric scaling is not used. The exact approach should be negotiated with the competent authorities.

The 100 x allometric scaling is also in agreement with FDA’s “Guidance for industry, Developing medical imaging drugs and biological products, Part 1: conducting safety assessments”:

“We recommend that the NOAEL in safety pharmacology studies in suitable animal species be at least one hundred times (100x) greater than the maximal mass dose to be used in human studies.”

It is recommended for all approaches to use a body weight of 50 kg for a patient to be on the safe side.

A major limitation of this microdosing approach is that it does not take into account that pharmacological and toxicological effects are usually not determined by the mass but the molar amount to be applied. For larger molecules such as peptides or proteins this causes a major limitation of the microdosing concept based solely on the mass. It should be noted, that the average molecular weight (M_w_) of a drug compound is approx. 300 g/mol.

Therefore, from a pharmacological point of view a peptide with a M_w_ of approx. 1500 g/mol having the same pharmacological potency as a low molecular weight drug could be used in a five-fold higher amount. In such a case the microdosing concept could be extended to, e.g., <500 μg and still be within the same equimolar limit. For larger molecules such as proteins the FDA’s “Guidance for Industry, Investigators, and Reviewers; Exploratory IND Studies” sets the limit to <30 nmoles: “Due to differences in molecular weights as compared to synthetic drugs, the maximum dose for protein products is ≤30 nanomoles.” This corresponds to <100 μg of a drug having a molecular weight of 300 g/mol.

It also should be noted that for radiopharmaceuticals, usually extensive biodistribution data (often including imaging) from preclinical studies are available. These studies give detailed quantitative data on accumulation and elimination in tissues and excretion pathways. Based on such data the design of extended single dose toxicity studies may be focused on risk organs and tissues, thereby eliminating the requirement especially for (high cost) histopathological data in all organs, focusing on main organs where the RP accumulates by, e.g., binding to receptors or other target structures. Such design has to be made on a case-by case basis and its rational being described in detail in the application process.

### >100 μg

Applications of more than 100 μg substance in the case of RPs may be required for imaging with peptides and proteins or for therapeutic applications. The assumptions made focus on single dose applications in clinical studies, i.e., the RP is expected to be cleared completely from the blood before the next application.

#### For imaging

In this case, also the “Note for guidance on non-clinical safety studies for the conduct of human clinical trials and marketing authorization for pharmaceuticals” can be applied using approach 3. This includes also an extended single dose toxicity study, however, in this case in rodent and non-rodent species, as well as a test for genetoxicity (usually Ames test). Selection of histopathology data may be based on the same considerations as described in the <100 μg group. The toxicology tests must be performed in compliance with Good Laboratory Practice (GLP).

#### For therapy

For the anticancer use of RPs (immunoradiotherapy, endoradiotherapy) the “ICH guideline S9 on nonclinical evaluation for anticancer pharmaceuticals” can be employed. In these cases in particular genetoxicity testing for Phase I and II studies may not be required.

In accordance with scenario in section <100 μg, the argument of molar equivalence may be used.

## Conclusion

The concept of differentiating 3 toxicological limits will clarify the requirements for application of clinical trials of new radiopharmaceuticals and allow much better planning of translational studies, thereby strengthening research efforts towards novel targeting agents for molecular imaging and molecular radiotherapy. It is our aim to have clear recommendations from competent authorities, such as EMA, for the requirements on toxicological studies for radiopharmaceuticals. In the future we recommend that the mass limits will be replaced by molar amounts as this makes more sense from a pharmacological perspective and would facilitate (radio)pharmaceutical development and practice.

The guidelines give some general recommendations for necessary toxicological studies. But it is necessary to make a risk assessment for each compound to evaluate which toxicological studies are needed and useful. Therefore a scientific advice meeting with the competent authorities would be very helpful before starting expensive toxicity studies.

## Abbreviations

(Q)SAR: (Quantitative) structure-activity relationship
API: Active pharmaceutical ingredient
CHMP: Committee for Medicinal Products for Human Use
CPMP: Committee for proprietary medicinal products
EMA: European Medicines Agency
GLP: Good laboratory practice
ICH: International Conference on Harmonisation of Technical Requirements for Registration of Pharmaceuticals for Human Use
MTD: Maximum tolerated dose
NOAEL: No observed adverse effect level
NOEL: No observed effect level
PET: Positron emission tomography
SCCP: Scientific committee on consumer protection
SWP: Safety working party
TTC: Toxicological threshold of concern
